# Invasive fungal infection by *Cryptococcus neoformans* var. *grubii* with bone marrow and meningeal involvement in a HIV-infected patient: a case report

**DOI:** 10.1186/s12879-019-3831-8

**Published:** 2019-03-04

**Authors:** Hareton Teixeira Vechi, Raquel Cordeiro Theodoro, Andrea Lima de Oliveira, Ronald Muryellison Oliveira da Silva Gomes, Rodolfo Daniel de Almeida Soares, Munya Gandour Freire, Mônica Baumgardt Bay

**Affiliations:** 1 Department of Infectious Diseases, Hospital Giselda Trigueiro, Universidade Federal do Rio Grande do Norte, 110 Cônego Monte Street, Natal, Rio Grande do Norte RN 59037-170 Brazil; 2 Department of Celular Biology and Genetics/Institute of Tropical Medicine, Universidade Federal do Rio Grande do Norte, Natal, Rio Grande do Norte RN 59078-970 Brazil; 3 Department of Hematology, Universidade Federal do Rio Grande do Norte, 619ª Nilo Peçanha Avenue, Natal, Rio Grande do Norte RN 59012-300 Brazil; 4 Department of Pathology, Hospital Universitário Onofre Lopes, 600 Nilo Peçanha Avenue, Natal, Rio Grande do Norte RN 59012-300 Brazil

**Keywords:** Cryptococcosis, *Cryptococcus neorformans*, AIDS, HIV, Bone marrow

## Abstract

**Background:**

Cryptococcosis is a common opportunistic infection in patients infected by Human Immunodeficiency Virus (HIV) and is the second leading cause of mortality in Acquired Immunodeficiency Syndrome (AIDS) patients worldwide. The most frequent presentation of cryptococcal infection is subacute meningitis, especially in patients with a CD4+ T Lymphocytes count below 100 cells/μL. However, in severely immunosuppressed individuals *Cryptococcus neoformans* can infect virtually any human organ, including the bone marrow, which is a rare presentation of cryptococcosis.

**Case presentation:**

A 45-year-old HIV-infected male patient with a CD4+ T lymphocyte count of 26 cells/μL who presented to the emergency department with fever and pancytopenia. Throughout the diagnostic evaluation, the bone marrow aspirate culture yielded encapsulated yeasts in budding, identified as *Cryptococcus* sp. The bone marrow biopsy revealed a hypocellularity for age and absence of fibrosis. It was observed presence of loosely formed granuloma composed of multinucleated giant cells encompassing rounded yeast like organisms stained with mucicarmine, compatible with *Cryptococcus* sp. Then, the patient underwent a lumbar puncture to investigate meningitis, although he had no neurological symptoms and neurological examination was normal. The cerebrospinal fluid culture yielded *Cryptococcus* sp. The species and genotype identification step showed the infection was caused by *Cryptococcus neoformans* var. *grubii* (genotype VNI). The patient was initially treated with amphotericin B deoxycholate plus fluconazole for disseminated cryptococcosis, according to guideline recommendations. However, the patient developed acute kidney injury and the treatment was switched for fluconazole monotherapy. The symptoms disappeared completely with recovery of white blood cells and platelets counts. Cerebrospinal fluid cultures for fungi at one and two-weeks of treatment were negative.

**Conclusions:**

Bone marrow infection caused by *Cryptococcus neoformans* is a rare presentation of cryptococcosis. The cryptococcal infection should be included for differential diagnosis in HIV-infected patients with fever and cytopenias, especially when CD4+ T lymphocytes count is below 100 cells/μL.

## Background

Cryptococcosis remains the second most frequent cause of death in AIDS patients worldwide, only behind tuberculosis [1]. Its most frequent presentation is subacute meningitis, which ranges from 70 to 90% of cases [2], usually in patients with CD4+ T lymphocyte count bellow 100 cells/μL [3]. Recent estimates suggest that about 223,000 new cases of cryptococcal meningitis are diagnosed every year, causing 181,000 deaths [1]. Cases usually occur in patients who are unaware of their HIV infection, being cryptococcosis their AIDS-defining illness, or in those with limited access to antiretroviral therapy [4]. In both situations, cryptococcal infection indicates a failure in AIDS control programs.

In turn, HIV-infected patients with severe immunodeficiency commonly present fever accompanied with cytopenias. The hematological abnormalities can be due to HIV infection itself or non-infectious and infectious causes. The most frequent opportunistic infections that cause pancytopenia are histoplasmosis and mycobacteriosis [5]. In severely immunosuppressed patients, *Cryptococcus neoformans* can infect virtually any human organ system, including the bone marrow, which is a rare presentation of cryptococcosis.

Here, we describe an uncommon presentation of disseminated cryptococcosis with invasive disease of bone marrow and asymptomatic meningitis.

## Case presentation

A 45-year-old HIV-infected black man, mechanic, has sought emergency department referring progressive asthenia for two weeks, with difficulty on performing basic daily life activities, anorexia, fever and profuse sweating. During this time, he reported a weight loss of 3Kg. In the last 24 h, he had vomiting episodes preceded by nausea, in small volume, without relation to feeding. The patient denied other symptoms like headache, cough, abdominal pain and diarrhea. The HIV infection was diagnosed 8 years ago and his current antiretroviral therapy consisted of tenofovir disoproxil fumarate – lamivudine – efavirenz in a once-daily single-pill. His medical history was remarkable for poor adherence to antiretroviral therapy. His recent CD4+ and CD8+ T lymphocytes counts were 26 (1.92%) and 509 (37.9%) cells/μL respectively and the viral load was 252,624 copies/mL (5.402 Log_10_). He was also using a trimethoprim-sulfamethoxazole double-strength tablet for *Pneumocystis jirovecii* prophylaxis. The patient had pulmonary tuberculosis 8 years ago, his AIDS-defining illness. In addition, he had several previous hospitalizations for chronic diarrhea. He used to drink distilled beverages thrice a week and denied tobacco use. The patient raised a dog and a parakeet as pets. He was born in Angicos City, rural area of Rio Grande do Norte, Brazil, an endemic region for visceral leishmaniasis and Chagas disease.

On admission, vital signs were: axillary temperature 38.0 °C, blood pressure 100/80 mmHg, pulse rate of 110 bpm and respiration rate of 24 bpm. His physical examination was remarkable for cachexia (body weight of 38Kg and body mass index of 13.9Kg/m^2^) and a mild hepatomegaly. He was lucid and oriented. There were no signs of meningeal irritation. The neurological examination was normal. Laboratory tests showed pancytopenia: hemoglobin of 100 g/L; white blood cell counts of 2.7 × 10^9^/L (74% neutrophils and 22% lymphocytes) and platelets count of 123 × 10^9^/L. The erythrocyte sedimentation rate was 68 mm [Reference Interval (RI): 0 – 15 mm] and serum C - reactive protein level was 28.5 nmol/L (RI: < 57.1 nmol/L). The serum creatinine was 0.058 mmol/L (RI: 0.044–0.106 mmol/L) and serum urea was 3.18 mmol/L (RI: 1.66–8.32 mmol/L). The alanine and aspartate aminotransferases levels were 43 and 23 IU/L (RI: 0–37 and 0 – 42 IU/L, respectively). The alkaline phosphatase and gamma-glutamyl transferase levels were 201 IU/L and 49 IU/L (RI: 80 – 306 IU/L and 11 – 61 IU/L, in this order). The serum albumin was 39.4 g/L (RI: 35 – 55 g/L), total bilirubin level was 8.55 μmol/L (RI: 3.42–20.52 μmol/L) and prothrombin time and activity were 13.5 s and 100% (RI: 9.5–13.5 s and ≥ 70%). The serum lactate dehydrogenase level was 477 IU/L (RI: 225 - 450 IU/L). Chest X-ray was normal. An abdominal ultrasonography showed hepatomegaly and cholelithiasis.

He has been treated for febrile neutropenia with cefepime for 7 days, without changes in clinical symptoms. Blood cultures were negative for bacteria and fungi. The serologic tests were negative for hepatitis B and C, visceral leishmaniasis and Chagas disease. Tuberculin Test was non-reactive. Venereal disease research laboratory (VDRL) testing was negative too. Cryptococcal antigen testing in serum was not performed, because it is not available at our service. *Histoplasma* antigen testing in urine was negative. Due to pancytopenia, the patient underwent a bone marrow aspirate and biopsy. The direct examination presented a non-specific hypocellularity and was negative for fungi, mycobacteria and hemoparasites. After 5 days, bone marrow aspirate culture yielded encapsulated yeasts in budding on India ink stain, identified as *Cryptococcus* sp. Then, the patient underwent a lumbar puncture to investigate meningitis. The cerebrospinal fluid (CSF) had a clear appearance, an opening pressure of 160mmH_2_O, a cell count of 2 leukocytes, protein of 44 mg/dL and glucose of 55 mg/dL, and numerous encapsulated yeasts in budding were seen on India ink stain. The CSF culture yielded *Cryptococcus* sp. Bone marrow aspirate culture was negative for bacteria, mycobacteria and *Leishmania* sp.

**Fig. 1:**
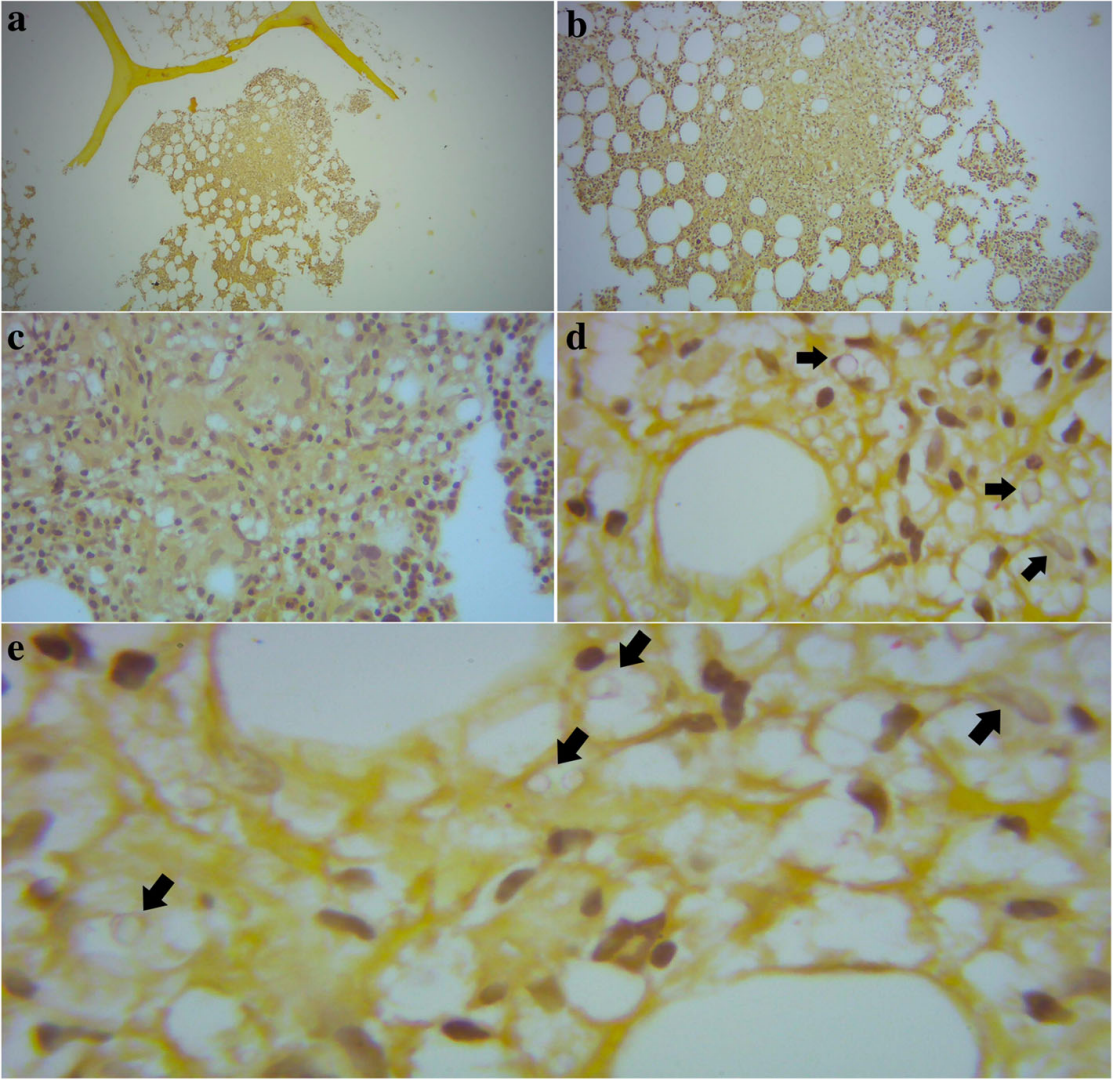
Photomicrography of the patient’s bone marrow biopsy. Effacement of normal bone marrow architecture at 40x and 100x magnification (**a** and **b**, respectively), with replacement of normal adipose tissue by an inflammatory infiltrate consisting of xanthomatous histiocytes and multinucleated giant cells, constituting a loosely formed granuloma, better visualized at 400x magnification (**c**). At this magnification, the multinucleated giant cells exhibit clear areas inside their cytoplasm. At 1000x magnification, such clear areas revealed to be rounded yeast like organisms (arrows), located inside multinucleated giant cells and histocytes, stained with mucicarmine in their capsule, compatible with *Cryptococcus* sp. (**d** and **e**)

The bone marrow biopsy revealed 30 % of hematopoietic cellularity with representation of the three cellular lineages, characterizing a hypocellular bone marrow for age. The megakaryocytes were present in increased numbers and megakaryocytes with mild dysplasia (hypolobated nuclei) were rarely observed. The myeloid series presented a preserved maturation scale with predominance of mature forms and the erythroid lineage was arranged in small islands without particularities. There were typical plasma cells scattered in hematopoietic tissue. Silver staining revealed a delicate network of reticulin fibers without fibrosis. The Masson’s trichrome staining showed no collagen fibers deposition. Giemsa, Fite-Faraco and Periodic Acid-Schiff staining were negative. In the mucicarmine staining, it was observed presence of loosely formed granuloma composed of multinucleated giant cells encompassing rounded yeast like organisms compatible with *Cryptococcus* sp. (Fig. 1).

**Fig. 2:**
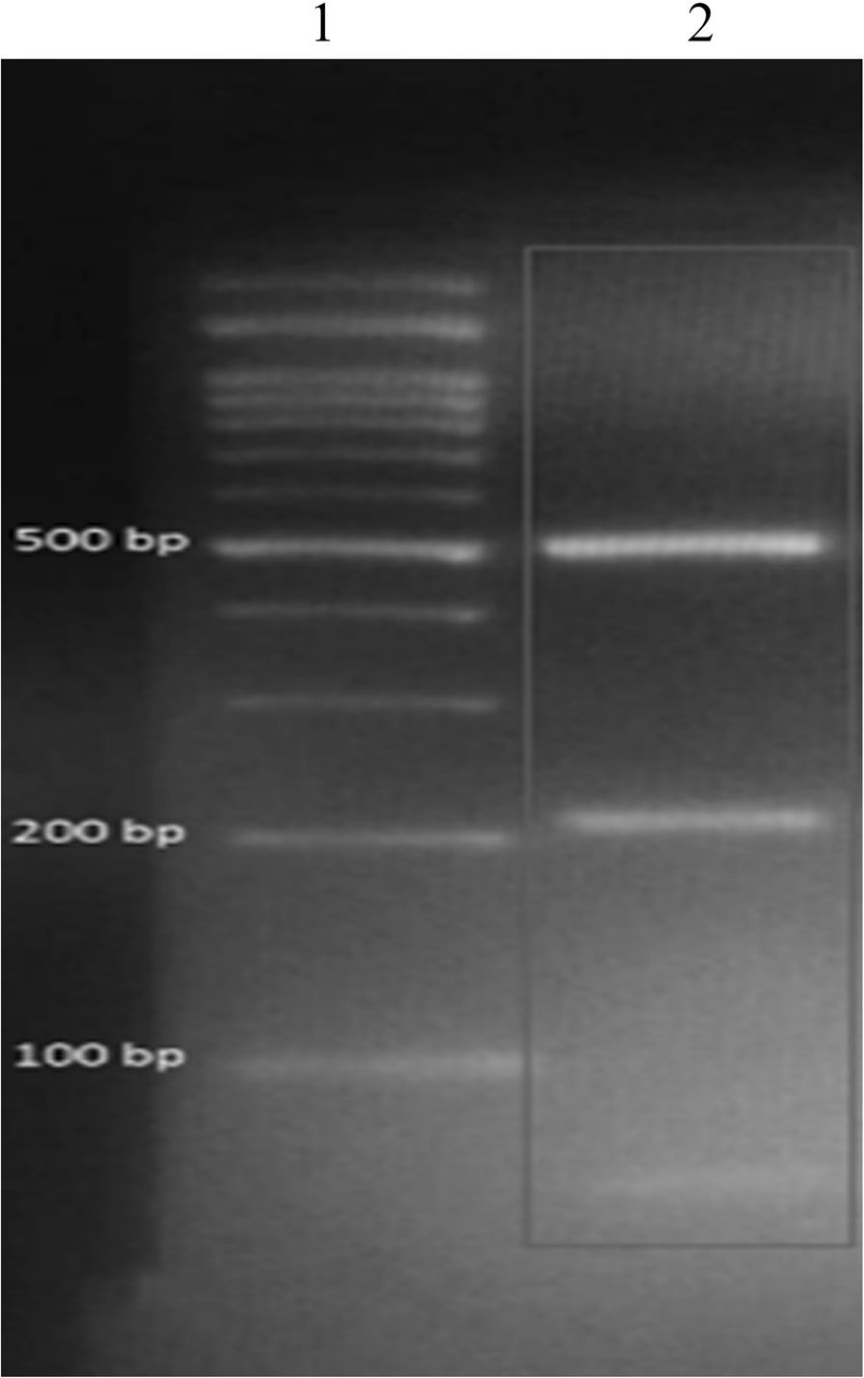
Genotyping by PCR-RFLP of *URA5* gene of *Cryptococcus neoformans* isolated from patient’s bone marrow culture. Lane 1: 100pb DNA ladder. Lane 2: fragments obtained after double digestion of *URA5* PCR products

For species and genotype identification, the isolate was grown on Sabouraud dextrose agar at 37 °C for 48 h and deoxyribonucleic acid (DNA) was extracted by initial mechanical rupture of yeast cell in liquid nitrogen using a pestle and mortar following the extraction protocol of McCullough et al. [6]. The *URA5* gene was amplified by Polymerase Chain Reaction (PCR) according to the method suggested by Meyer et al. [7]. The isolate was identified as *C. neoformans* var. *grubii* (genotype VNI) (Fig. 2).

The patient was treated with amphotericin B deoxycholate at 1 mg/Kg daily, combined with fluconazole at 800 mg daily given intravenously. After a week of treatment, the patient developed acute kidney injury stage 3, according to the Kidney Disease: Improving Global Outcomes (KDIGO) criteria [8], with increase of serum creatinine and urea levels to 0.185 mmol/L and 11.98 mmol/L, respectively. At the discretion of assistant doctor, the treatment was switched for fluconazole at 1200 mg daily during the remainder of the 14-day induction phase. The patient was conservatively managed for acute kidney injury with return of baseline serum creatinine and urea levels after five weeks. CSF cultures for fungi were negative at one and two-weeks of treatment. After that, the patient was treated with fluconazole at 800 mg daily throughout the 8-week consolidation phase.

The patient remained hospitalized throughout the induction and consolidation phases of the treatment due to socioeconomic reasons. He could not afford the costs related to the hospitalization neither his medicines, that was why the patient went to our hospital, a public health facility, besides he did not have an appropriate family support to help him recover and manage his condition. Cranial computed tomography (CT) showed cerebellar atrophy with compensatory fourth ventricle ectasia and cerebral atrophy with non-hypertensive dilation of the supratentorial ventricles and enlargement of grooves, fissures and cisterns. Magnetic resonance imaging (MRI) confirmed a non-habitual volumetric reduction of encephalic parenchyma for age with compensatory ectasia of ventricular system; the brain parenchyma signal-intensity was preserved. The patient had no fever nor vomiting within the first week of treatment and recovered from asthenia after four weeks of treatment. After induction and consolidation phases of treatment, the hematological parameters have improved: hemoglobin level of 110 g/L; white blood cell counts of 5.9 × 10^9^/L (65% neutrophils and 30% lymphocytes) and platelets count of 166 × 10^9^/L. He was discharged from hospital with recommendations to keep oral fluconazole at 450 mg daily (maintenance phase) and outpatient follow-up at a regional HIV/AIDS care service.

## Discussion and conclusions

The most frequent clinical manifestation of cryptococcosis in HIV-infected patients is subacute meningitis. Due to the neurotropism, the isolation of Cryptococcus sp. in extra-neural sites should prompt to a lumbar puncture to rule out concomitant meningitis [9]. In the present case, a lumbar puncture was justified after the detection of *Cryptococcus neoformans* in bone marrow aspirate culture, although the patient did not demonstrate clinical evidences of central nervous system (CNS) involvement. The finding of encapsulated yeasts in CSF was incidental, characterizing a subclinical or latent form of cryptococcal meningitis (CM).

Subclinical CM has previously been reported. Liss and Rimland [10] described two cases of asymptomatic CM. One patient was a 59-year-old diabetic man who was hospitalized for cryptococcal pneumonia. His neurological and ocular examinations were unremarkable and neck stiffness was absent. Blood cultures for fungi were negative. The urine culture yielded *Cryptococcus neoformans*. He was submitted to a lumbar puncture, with numerous encapsulated yeasts observed in the CSF, subsequently identified as *Cryptococcus neoformans*.

In a series of 40 cases of CM, Butler and colleagues [11] found four (10%) cases of asymptomatic CM after isolation of *Cryptococcus neoformans* in culture of extra-neural sites specimen: kidney, blood, lung, and a submandibular nodule. All patients had no symptoms of meningeal involvement, but one patient has been undergoing psychiatric treatment for personality change of several years duration. Smilack et al. [12] reported a case of a 21-year-old man with widespread Hodgkin’s lymphoma in remission with pleural effusion of cryptococcal etiology. He had no neurological symptoms, but *Cryptococcus neoformans* was also isolated in CSF culture.

The studies of the prevalence of cryptococcal antigenemia in HIV-infected patients with CD4 + T lymphocyte count below 100 cells/μL demonstrated the CM may be common in clinical practice. In a recently published meta-analysis, Temfack et al. [13] found a prevalence of 33% (95% confidence interval, 21–45%) of asymptomatic CM among cryptococcal antigen (CrAg) positive patients. The CrAg testing has been found to be a valuable tool in the early detection of cryptococcal infection in at-risk patients, as CrAg can be detected in blood weeks to months before the onset of CM [14]. Since 2011, World Health Organization has recommended routine CrAg testing in antiretroviral-naïve HIV-infected adult patients with CD4 + T lymphocyte count below 100 cells/μL, by either latex agglutination or lateral flow assay, followed by a targeted pre-emptive fluconazole therapy among CrAg-positive patients [15].

With pre-emptive fluconazole therapy, the incidence of CM dropped from 21.4 to 5.7% among CrAg-positive patients and the all-cause mortality reduced from 39.7 to 17.4%, although it still remained significantly higher among CrAg-positive than CrAg-negative patients (around two-fold risk) [13]. Besides blood, CrAg can be also detected in CSF and has been proved to be a rapid and reliable non-culture-based diagnostic test for confirmed CM in AIDS patients [16]. CSF CrAg testing is handy for timely diagnosis of CM, as it can be detected before the direct microscopic examination using India Ink is positive, whose sensibility is around 86%, while pending the CSF culture results [17]. Even CSF CrAg may be positive in occasional cases when the culture is negative due to previous exposure to antifungal therapy or the incubation period was not long enough, especially in low yeast burden associated with small volumes of CSF [17].

Unfortunately, the CrAg testing is not available at our service. However, it is worth pointing out that our patient was at high risk for cryptococcosis (CD4+ T lymphocytes count of 26 cells/μL) and would probably benefit from CrAg testing in blood for early detection of the cryptococcal infection, and once being positive, the CSF testing would be advantageous for early and timely diagnosis of meningeal involvement.

As the course of CM is usually indolent over weeks and symptoms and signs may be subtle and non-specific, a high degree of clinical suspicion is required, especially in patients with advanced HIV infection. Vomiting and difficulty on performing basic daily activities, being non-specific, might be interpreted as neurological symptoms for this patient. In CM, vomiting is often a clinical manifestation of elevated intracranial pressure (ICP). In addition, hydrocephalus, a possible complication of CM and a cause of elevated ICP, may provoke dementia symptoms with impairment of daily activities. However, the opening pressure of CSF on lumbar puncture was normal and neuroimaging exhibited a normal brain parenchyma sign and excluded cerebral oedema or hydrocephalus as causes of elevated intracranial pressure.

A possible explanation for the asymptomatic meningitis in our patient would be the early diagnosis of CNS involvement. The finding of *Cryptococcus neoformans* in bone marrow aspirate prompted us to investigate the CNS before the symptoms appeared. Perhaps the atrophy of the brain parenchyma observed in the patient’s neuroimaging studies may have contributed to the absence of symptoms slowing the process of increasing ICP. The patient’s opening pressure of CSF was 160mmH_2_O. Despite this, the pathophysiology related to the symptoms and signs of CM is not fully elucidated.

In the study by Graybill et al. [18], clinical manifestations of raised ICP, as headache, were more common in patients with higher pressures, nevertheless they were absent in up to 8% of patients with the highest pressures (≥ 350mmH_2_O) as well as 79% of patients with normal ICP (≤190mmH_2_O) reported headache. In cryptococcal meningitis, elevated ICP is caused by impaired CSF reabsorption at the arachnoid granulations secondary to an inflammatory arachnoiditis or direct cryptococcal infiltration [19]. Shankar et al. [20] raised the hypothesis of impedance of CSF circulation caused by masses of cryptococci on the arachnoid villi leading to dynamic hydrocephalus attacks, which are not recognized by routine methods as cranial CT or MRI in majority of instances.

In addition to CNS infections, HIV-infected patients also have more frequently disseminated cryptococcosis, with the finding of yeasts in extra-neural sites, associated to poorer prognosis [3, 4, 9, 11]. *Cryptococcus neoformans* has been reported to cause infection in any organ of the human body, including the bone marrow. In HIV-positive patients with disseminated cryptococcosis, the bone marrow involvement is observed from 13 to 42% of cases [4, 11, 21], with a wide spectrum of clinical manifestations.

Bone marrow infection by *Cryptococcus neoformans* can be absolutely asymptomatic in HIV-infected patients, and has been identified through screening cultures [21]. It can be an exclusive affection of bone marrow and present nonspecific symptoms like fever, anorexia, asthenia and weight loss as part of a wasting or fever of unknown origin syndrome, followed by pancytopenia [22]. In extreme cases, cryptococcosis can mimic aplastic anemia, with bone marrow biopsy showing almost complete acellularity in context of a disseminated disease [12].

The direct examination of bone marrow aspirate can show extra- and intracellular encapsulated yeast like organisms in budding or not, offering the advantage of a rapid turn-around time, while pending culture and biopsy results [22–24]. In biopsy study, the most common findings are the presence of granulomas with spherical encapsulated yeast [10, 22, 25], similar to direct examination. Specific stains as India ink, mucicarmine or Gomori methenamine silver are necessary for demonstration of the capsule and confirm the diagnosis of cryptococcosis [22–24]. The isolation of *Cryptococcus neoformans* from tissue culture adds to diagnosis.

Here in Rio Grande do Norte, a state of Northeast of Brazil, there are other more frequent opportunistic causes of pancytopenia in patients with AIDS as visceral leishmaniasis, disseminated histoplasmosis and tuberculosis. Thus, even though pancytopenia may result from cryptococcosis, the diagnosis was completely unexpected. Our patient has fulfilled the criteria for invasive fungal infection of bone marrow by *Cryptococcus neoformans*. The culture of bone marrow aspirate for fungi requires a volume of 1 to 5 mL. Collecting this volume leads to an unavoidable dilution of bone marrow aspirate with peripheral blood [26]. The yield of *Cryptococcus* sp. from this culture may result from: the true presence of yeast in bone marrow tissue; the presence of yeasts in peripheral blood (as part of cryptococcaemia) or both. In this case, the peripheral blood culture for fungi, incubated for 8 weeks, as part of diagnostic work-up of disseminated histoplasmosis, was negative. Moreover, the presentation of granulomatous reaction with encapsulated yeasts inside multinucleated giant cells is an unequivocal evidence of bone marrow infection by *Cryptococcus* sp.

In immunocompromised patients, the cryptococcal disease may result from either primary infection or reactivation of a latent infection. There are epidemiological and biological evidence of the role of quiescence on pathogenesis of cryptococcosis [27, 28]. In study by Saha et al. [29], the transplant recipients with previous serological evidence of cryptococcal infection (latent infection) developed cryptococcosis earlier after transplant (5.6 ± 3.4 months) than patients without preexisting serological reactivity (40.6 ± 63.8 months), suggesting an acute infection in the latter group. In this case, the patient was not very adherent to antiretroviral therapy and his CD4+ T Lymphocyte count has been below 100 cells/μL for around 6 years (data not showed).

Furthermore, this patient had a remarkable epidemiological record of parakeet exposure. *Cryptococcus neoformans* has been isolated from the droppings of a variety of avian species [30]. Although a history of bird exposure is not commonly evoked from patients with cryptococcosis, there are documented cases of probable transmission of *Cryptococcus* sp. from exposure to aerosolized birds excrements to immunocompromised patients, suggesting a progressive primary infection [31, 32]. Taking into account the parakeet exposure and presentation of cryptococcosis after about 6 years of advanced HIV infection, we can speculate this patient might have developed a disseminated cryptococcosis by primary infection.

In disseminated cryptococcosis, the recommended treatment consists of an induction phase with an amphotericin B formulation given intravenously combined with oral flucytosine for, at least, two weeks. This combination is associated with more rapid sterilization of CSF and better survival [33]. Unfortunately, we do not have flucytosine in our service. Thus, amphotericin B formulation combined with fluconazole at 800 mg daily is a viable option in this circumstance.

Liposomal amphotericin B is preferable to conventional formulation, since it shows similar efficacy, but less toxicity [34]. Our patient developed acute renal failure after a week of treatment with amphotericin B deoxycholate. At discretion of assistant doctor, treatment was switched for fluconazole at 1200 mg daily, according to guidelines recommendations [33]. The patient had a good response to the treatment. CSF cultures for fungi at one and two-weeks were negative. The symptoms disappeared completely with recovery of leucocytes and platelets counts. In the maintenance therapy of CM, fluconazole is used to prevent relapses until an adequate immunological recovery. Our patient had an advanced HIV disease, as evidenced by his extremely low CD4 count, and several predictors of poor outcome of cryptococcal infection: a low baseline body weight, a poor baseline CSF inflammatory response (< 20cells/μL of CSF), a low Karnofsky performance status at presentation and a disseminated disease with positive extraneural culture [35]. Taking into account these prognostic factors, at the decision of assistant doctor, the patient used a higher than usual dose of fluconazole (200 mg daily) in the maintenance phase, based on the Brazilian guidelines for management of cryptococcosis [33, 36].

In conclusion, bone marrow infection caused by *Cryptococcus neoformans* is uncommon. This etiology should be included for differential diagnosis in HIV-infected patients with fever and cytopenias, especially when CD4 T lymphocytes count is below 100 cells/μL. This case illustrates an atypical presentation of disseminated cryptococcosis with bone marrow involvement and asymptomatic meningitis, highlighting the role of bone marrow aspirate and biopsy study in HIV-infected patients with fever and pancytopenia for early diagnosis and adequate treatment of opportunistic infections.

## Abbreviations

AIDS: Acquired Immunodeficiency Syndrome
CM: Cryptococcal Meningitis
CNS: Central Nervous System
CrAg: Cryptococcal Antigen
CSF: Cerebrospinal Fluid
CT: Computed Tomography
DNA: Deoxyribonucleic Acid
HIV: Human Immunodeficiency Virus
ICP: Intracranial Pressure
MRI: Magnetic Resonance Imaging
PCR: Polimerase Chain Reaction
RFLP: Restriction Fragment Length Polymorphism
RI: Reference Interval
VDRL: Venereal Disease Research Laboratory
VNI: *Cryptococcus neoformans* genotype I
